# From informal practice to structured care: pharmaceutical indication and minor ailment management in Latin American community pharmacy

**DOI:** 10.3389/fpubh.2026.1790792

**Published:** 2026-03-13

**Authors:** Esteban Zavaleta-Monestel, Jesús Carlos Gómez-Martínez, Fernando Martínez-Martínez, Audry Escudero-Correa, Lars-Ake Soderlund, Jeaustin Mora-Jiménez, Sebastián Arguedas-Chacón

**Affiliations:** 1 Health Research Department, Hospital Clinica Biblica, San Jose, Costa Rica; 2 Sociedad Científico Profesional de Farmacia Iberoamericana Comunitaria (SOCFIC), Barcelona, Spain; 3 Cátedra Atención Farmacéutica, Universidad de Granada, Granada, Spain; 4 International Pharmaceutical Federation (FIP), The Hague, Netherlands

**Keywords:** health policy, implementation science, Latin America, minor ailment management, pharmaceutical indication, primary health care, scope of practice

Persistent gaps in access to essential medicines and timely primary care continue to shape healthcare experiences across Latin America. These gaps endure despite the significant involvement of community pharmacies in addressing unmet health needs. Each day, millions rely on pharmacists for assistance with symptoms, treatment decisions, and safe medicine use. However, most health systems only weakly reflect these contributions in policy and regulation. The disconnect between pharmacists' practical roles and formal recognition by health systems is no longer a marginal concern. Instead, it reflects a longstanding decision to leave one of the most accessible points of care largely without guidance. As pressure on primary care intensifies, maintaining this situation is increasingly difficult to justify (1–3).

It is important to acknowledge that “Latin America” does not represent a homogeneous regulatory or organizational landscape. Community pharmacy ownership models, scope-of-practice definitions, professional oversight mechanisms, and integration with primary care vary substantially across countries. The patterns described in this article therefore reflect recurring regional tendencies rather than uniform national realities (2).

This disconnect is particularly notable given the central role community pharmacies occupy within the health landscape. Pharmacies are open, accessible, and integrated into daily life. In many urban neighborhoods, rural areas, and regions with limited physician access, pharmacies are often the initial point of contact for individuals seeking assistance. Pharmacists provide symptom assessments, recommend treatments, and advise when further medical attention is necessary. In this capacity, they frequently serve as intermediaries between patients and fragmented health systems. Evidence from low-resource settings consistently demonstrates that community pharmacies facilitate access to medicines and basic care when formal pathways are inaccessible or slow to respond (4–6).

Despite widespread reliance on community pharmacists, their clinical role in Latin America remains inadequately defined. Pharmacists routinely perform activities beyond dispensing, yet these functions are seldom supported by clear protocols, documentation standards, or formal integration with primary care services. Consequently, practice varies significantly between settings, even among pharmacies governed by the same regulations. This ambiguity leaves pharmacists professionally vulnerable and limits the capacity of health systems to utilize community pharmacy as a consistent care component. Studies of pharmacy practice and medicine use across the region consistently identify fragmentation, inconsistent oversight, and limited clinical governance as persistent weaknesses (7–9). These weaknesses are most apparent in the management of minor ailments. Common conditions such as mild pain, cough, allergic symptoms, gastrointestinal discomfort, and minor skin problems generate substantial healthcare demand, despite being largely self-limiting. When managed appropriately, these conditions do not require physician intervention. However, self-medication without professional guidance is associated with well-documented risks, including inappropriate treatment choices, dosing errors, harmful drug interactions, and delayed identification of more serious illnesses. In this context, pharmacists are well-positioned to mitigate harm, but only if their roles in assessment, recommendation, and referral are clearly defined and supported (10).

Where clear protocols exist, evidence from other health systems demonstrates that pharmacist-led management of minor ailments can be both safe and effective. Services structured around defined protocols and referral criteria consistently report favorable symptom resolution, high patient satisfaction, and reduced strain on other healthcare sectors (11–13). The distinguishing feature of these models is not merely expanded authority, but the establishment of agreed clinical processes, defined responsibilities, and appropriate training that enable pharmacists to act with confidence and consistency.

While clinical effectiveness and patient satisfaction have been consistently reported in structured minor ailment services, economic outcomes are less uniformly documented. Some studies from high-income settings suggest potential cost savings through reduced physician consultations and improved allocation of primary care resources, particularly when remuneration mechanisms are aligned with service delivery (14). However, the transferability of such economic findings to Latin American contexts remains uncertain, given differences in financing structures, reimbursement systems, and workforce distribution. Economic sustainability should therefore be considered an essential component of future regulatory reform, rather than assumed as an automatic consequence of role formalization.

In Latin America, however, practices resembling pharmaceutical indication already form part of routine community pharmacy work, but without this level of structure. Studies from the region describe pharmacists recommending medicines for specific symptoms and helping patients decide when further care is needed (15, 16). Yet this activity remains largely informal. In many cases, it is tolerated rather than formally acknowledged, leaving pharmacists working in a gray zone between professional responsibility and regulatory uncertainty. Without explicit recognition and governance, pharmaceutical indications can easily be misunderstood as boundary-crossing rather than recognized as pragmatic responses to gaps in access. Any move toward formal recognition must therefore be accompanied by clear protocols, documentation expectations, and defined competencies.

At the same time, data on pharmacists' and population perceptions regarding such regulatory evolution in Latin America remain limited. Understanding levels of professional readiness, perceived benefits, and potential reluctance represents an important research gap and a critical determinant of successful implementation (17).

A relevant reference for this transition is found in the Ibero-American context, particularly in Spain and, to a lesser extent, Portugal. These countries share legal traditions, educational structures, and health system organizations familiar to much of Latin America. In Spain, pharmaceutical indication for minor ailments has evolved within structured clinical frameworks that emphasize assessment criteria, defined practice limits, documentation, and referral. Notably, this evolution did not require a fundamental redesign of the health system, but rather clarification of professional roles within existing regulatory structures. The focus has remained on standardization, accountability, and alignment with primary care, rather than on unrestricted expansion of scope. This experience also underscores the importance of aligning undergraduate education and continuing professional development with protocol-based practice, ensuring pharmacists are prepared for the responsibilities they already undertake (18).

This contrasts sharply with models from countries such as Canada and the United Kingdom. While pharmacist prescribing and formal minor ailment schemes in those settings have produced positive outcomes, they are embedded in regulatory, financial, and professional arrangements that differ substantially from those in Latin America. Attempting to replicate these approaches without adaptation risks creating misalignment between policy ambition and system capacity, reinforcing the need for solutions grounded in regional realities rather than imported wholesale (19, 20).

From an implementation science perspective, policy transfer without contextual adaptation frequently fails due to misalignment between intervention design and system readiness. Successful reform requires attention to local regulatory culture, interprofessional dynamics, financing mechanisms, and training capacity. Structured pharmaceutical indication in Latin America would therefore require iterative adaptation rather than direct replication of foreign models (21).

In this context, the primary risk facing community pharmacy practice in Latin America is not the expansion of pharmacists' clinical roles, but the persistence of unregulated variability. Pharmaceutical indication and minor ailment management already occur daily. Permitting these practices to continue without consistent standards, adequate training, and quality assurance exposes patients to variable care and erodes trust in the profession. This risk is exacerbated when pharmacies operate without a pharmacist present. Expecting pharmacies to provide clinical advice under such conditions undermines both safety and accountability. Consistent pharmacist presence is therefore not merely an operational preference, but a prerequisite for any credible effort to formalize or expand clinical services in community pharmacy (22, 23).

However, formalization of currently informal practices may also generate unintended consequences. Interprofessional tensions can emerge when expanded or clarified pharmacy roles are perceived as encroaching upon medical or nursing domains. Evidence from role theory and studies on pharmacist prescribing suggests that successful evolution of professional roles depends not only on regulatory change, but also on negotiated legitimacy, collaborative practice agreements, and shared understanding among health professionals. Proactively addressing these dynamics is essential to avoid resistance and fragmentation during implementation (24, 25).

Moving from informal practice to structured care also depends on professional leadership that extends beyond national boundaries. Across Ibero-America, the Sociedad Científico Profesional de Farmacia Iberoamericana Comunitaria (SOCFIC) has emerged as a regional space where community pharmacists can reflect collectively on shared challenges. By facilitating dialogue on pharmaceutical indication, minor ailment management, and the broader clinical role of community pharmacy, SOCFIC helps make visible the common patterns of role ambiguity and fragmentation that characterize the region. Such spaces are essential for developing a shared professional language and for aligning everyday practice with evolving policy frameworks (26).

Taken together, these considerations point toward the need for deliberate system choices. Strengthening community pharmacy practice in Latin America requires investment in education and continuing professional development aligned with protocol-based care, formal recognition of pharmaceutical indication as a legitimate clinical function, consistent pharmacist presence within community pharmacies, and the definition of graduated scopes of practice linked to competencies and accountability. These steps are not about professional expansion for its own sake. They reflect a realistic response to existing access gaps and a practical way to reinforce primary care through a workforce already embedded in communities. Continuing to avoid these decisions will not preserve safety or control; it will simply perpetuate informality, variability, and missed opportunities to improve care (14, 27).

Community pharmacies already contribute to addressing access challenges in Latin America. The central question is not whether pharmacists are involved in care, but whether health systems are prepared to govern this contribution in a manner that is safer, more consistent, and more transparent. This decision is no longer theoretical; it is actively influencing the delivery of care each day.
